# Central serous chorioretinopathy after scalp and eyebrow intralesional triamcinolone acetonide injections: Report of two cases

**DOI:** 10.1016/j.jdcr.2024.06.034

**Published:** 2024-07-14

**Authors:** Deesha Desai, Ambika Nohria, Lina Alhanshali, Michael Buontempo, Kristen I. Lo Sicco, Craig Fern, Jerry Shapiro

**Affiliations:** a University of Pittsburgh School of Medicine, Pittsburgh, Pennsylvania; b The Ronald O. Perelman Department of Dermatology, NYU Grossman School of Medicine, New York, New York; c Department of Dermatology, SUNY Downstate College of Medicine, Brooklyn, New York; d Department of Dermatology, Hackensack Meridian School of Medicine, Nutley, New Jersey; e Diseases & Surgery of the Retina, Mount Kisco, New York

**Keywords:** alopecia areata, central serous chorioretinopathy, corticosteroids, hair loss

## Background

Central serous chorioretinopathy (CSC) is a condition characterized by the accumulation of fluid under the retina secondary to serous retinal detachment or retinal pigment epithelial detachment. While the precise etiology is incompletely understood, it has been observed in conjunction with the use of systemic corticosteroids as well as with the application of local topical or intralesional corticosteroids. Notably, there have been no documented cases linking CSC to intralesional corticosteroid injections administered to the scalp. Herein, we present 2 cases where patients developed CSC subsequent to receiving intralesional cortisone injections on the scalp as a treatment for alopecia areata (AA).

## Report of two cases

**Fig 1:**
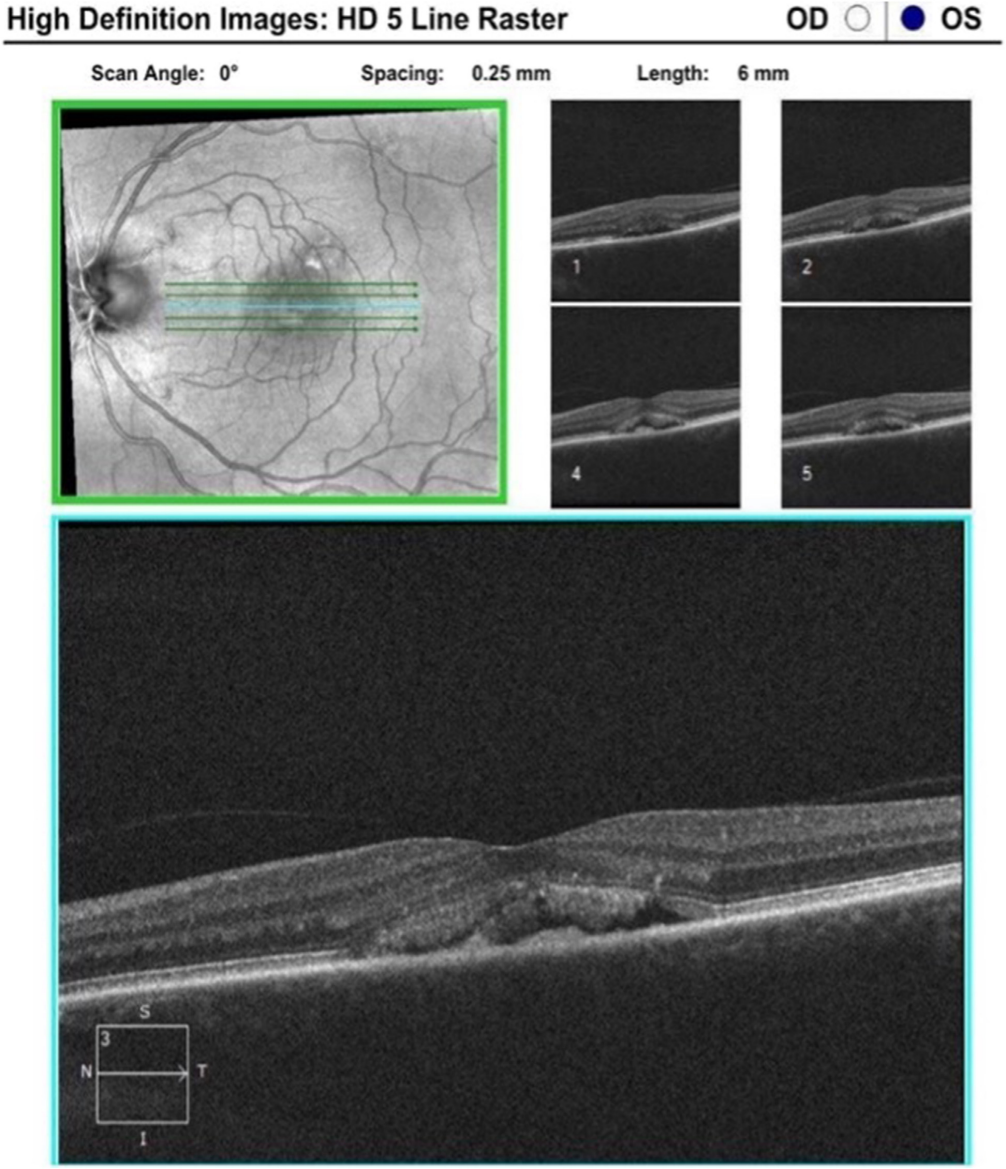
Optical coherence tomography (OCT) images (February 2022 [IL-TAC discontinued]).

**Fig 2:**
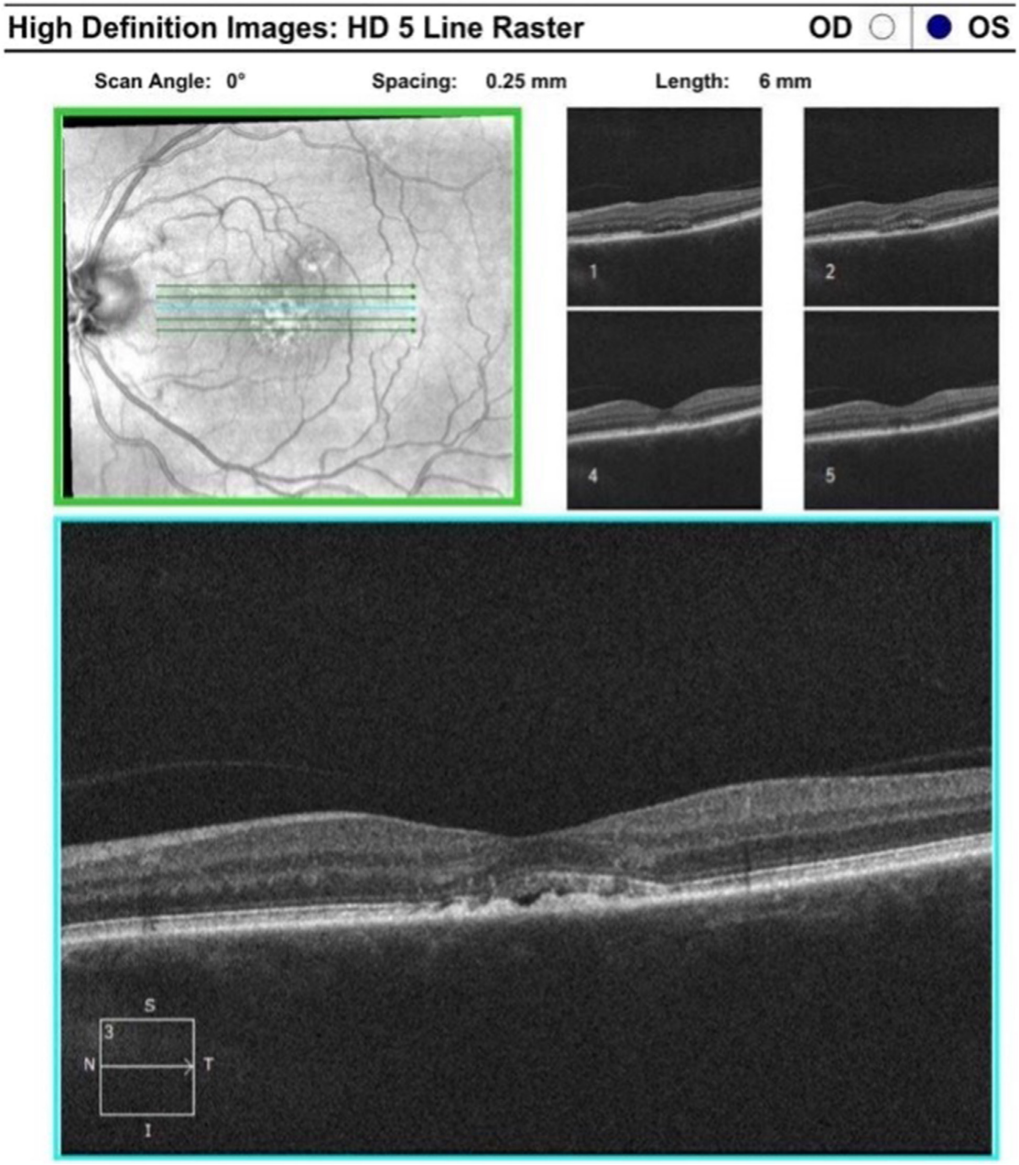
Optical coherence tomography (OCT) images (May 2022 [IL-TAC discontinued]).

**Fig 3:**
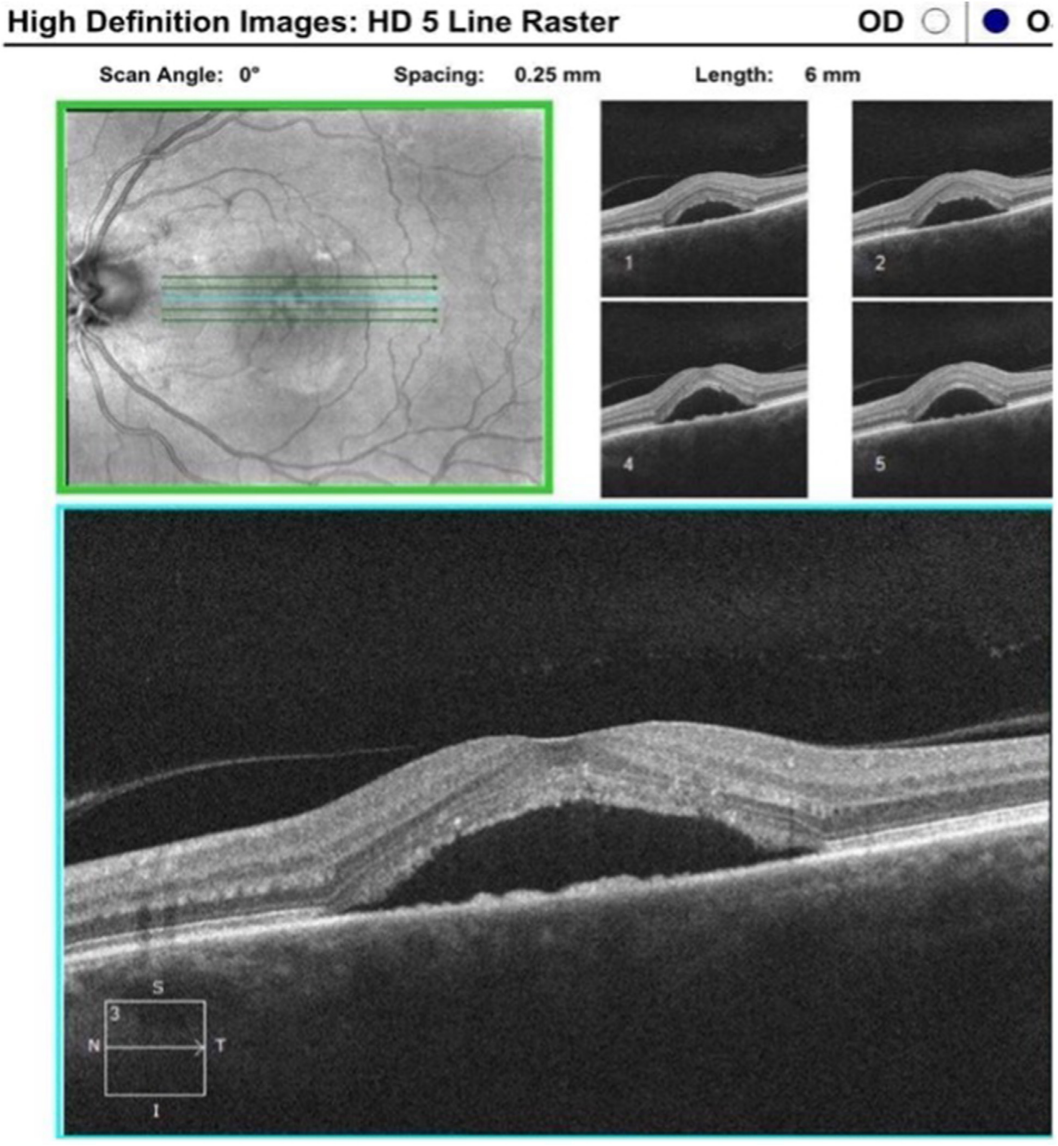
Optical coherence tomography (OCT) images (August 2022 [IL-TAC discontinued]).

**Fig 4:**
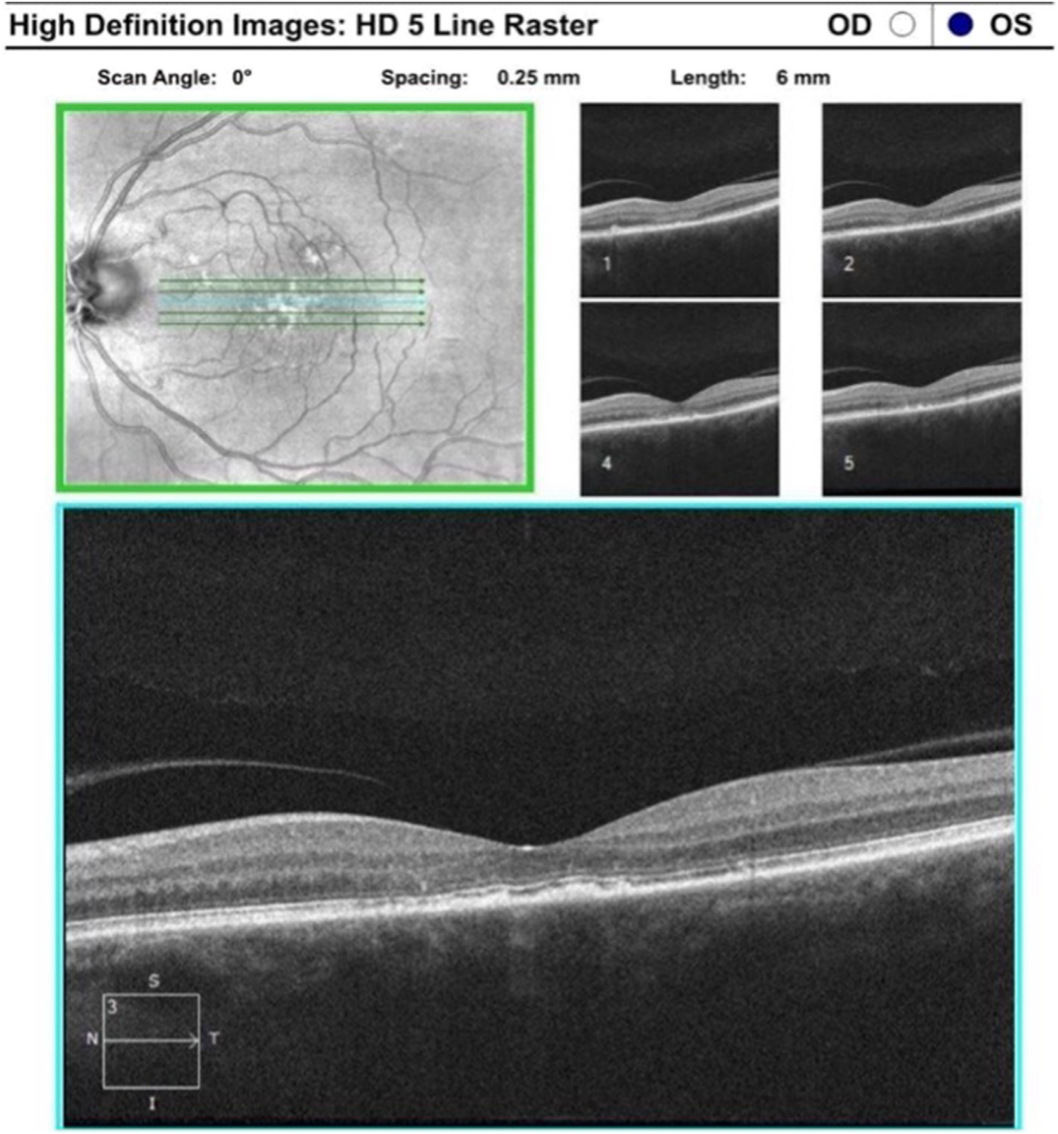
Optical coherence tomography (OCT) images (May 2023 [IL-TAC 10 mg over 4 mL, 5 treatments of reduced dose received]).

A 58-year-old female with no significant medical history presented to the dermatology clinic in June 2021 reporting diffuse hair loss. Upon physical examination, including trichoscopic assessment, findings were consistent with diffuse AA. Treatment commenced with monthly intralesional triamcinolone acetonide (IL-TAC) of 9 cc, 2.5 mg/cc across the scalp, along with topical applications of clobetasol 0.05% solution, and minoxidil 5% solution. The patient received 4 IL-TAC treatment sessions for 4 consecutive months and exhibited a notably favorable response. However, during an annual eye exam 6 months after the first IL-TAC treatment and 2 months after the last IL-TAC treatment, a retinal scan revealed fluid accumulation beneath the retina, raising concern for CSC. This diagnosis was confirmed by a retina specialist (C.F.) with close observation recommended. Due to this concern of CSC, IL-TAC was halted starting the month after the retinal scan was performed for the next 8 months (January to October 2022) (Figs 1 and 2). Following further consultation with the retina specialist, the IL-TAC dosage was adjusted down to 4 cc. The patient received 7 treatments at the reduced dose thereafter through the next 10 months (October 2022 to July 2023), with subsequent follow-up visits demonstrating improvement in CSC and stabilization of the condition since May 2023 (Figs 3 and 4). Regular retinal monitoring continues alongside ongoing IL-TAC injections. Notably, the patient did not report any ocular symptoms before, during, or after the CSC diagnosis. Annual ophthalmologic examinations were routine due to the patient’s family history of various ocular conditions, including retinal atrophy in the patient’s mother along with glaucoma, cataracts, macular degeneration, and cornea transplant in other relatives.

The second case involves a 34-year-old male presenting in December 2017 with AA. Treatment initiation included IL-TAC injections of 2.5 mg/mL, 3 mL to the scalp, alongside topical minoxidil 5% and clobetasol 0.05%. In October 2020, nearly 3 years after initial presentation, the patient developed new alopecic patches in the chin, beard, and eyebrows, for which IL-TAC injections of 2 cc, 2.5 mg/cc were administered every 3 months. In January and February 2022, 15 and 16 months later, the patient received 9 cc injections to the scalp and eyebrows, in addition to ongoing topical minoxidil, finasteride, and topical ruxolitinib. One month after this series of injections, experiencing intermittent blurry vision for a week, the patient sought consultation with an ophthalmologist and retina specialist, resulting in the diagnosis of CSC. Discontinuation of IL-TAC injections led to improvement in CSC symptoms. Notably, the patient had no personal history of ocular conditions, though his mother had a known history of cataracts.

## Discussion

CSC stands as the fourth most prevalent retinopathy, predominantly affecting young to middle-aged men, with a male-female ratio of 6:1.^1^ The condition manifests through serous retinal detachment and/or retinal pigment epithelial detachment, often leading to symptoms such as blurred vision, metamorphopsia, or micropsia.^2^ However, CSC may also be asymptomatic.

CSC is associated with elevated cortisol levels, stemming from various factors such as psychological stress, exogenous corticosteroid use, Cushing disease, or pregnancy.^2^ A meta-analysis highlighted a markedly increased risk of CSC in patients with prior corticosteroid usage compared to nonusers (OR 4.050, 5% CI 2.270 to 7.220, I2 = 59%, *P* < .001).^3^ The study further delineated that the risk was higher for corticosteroids administered orally, via injections, or nasally, in contrast to inhaled corticosteroids.

Numerous case reports have underscored the link between CSC development and exogenous corticosteroids administration through systemic, intravenous, intranasal, or topical routes.^4^, ^5^, ^6^ However, the timeline for CSC development after corticosteroid use remains unknown. In many instances, CSC resolved upon reducing or discontinuing steroid dosage. Notably, there have not been any documented cases of CSC following IL-TAC injections to the scalp or eyebrows. A singular case report has been documented regarding CSC onset following topical minoxidil application to the scalp.^7^ Interestingly, both patients in this report were using topical minoxidil. However, the temporal sequence of events, particularly the emergence of CSC subsequent to IL-TAC initiation or dose escalation and its subsequent resolution following dose reduction or discontinuation, suggests IL-TAC injections as the likely causative factor.

IL-TAC serves as a primary treatment option for patchy AA, which may necessitate injections to affected areas, including the eyebrows. This unexpected association underscores the need for clinicians to exercise caution when considering corticosteroid treatments, even in localized applications, and to remain vigilant for potential ocular complications such as CSC. Further research and monitoring are warranted to better understand the underlying mechanisms and risk factors involved in the development of CSC following scalp corticosteroid injections.

## Conflicts of interest

Dr Shapiro is a consultant for Aclaris Therapeutics, Incyte, and Replicel Life Sciences. Drs Shapiro and Lo Sicco have been investigators for Regen Lab and are investigators for Pfizer. Dr Lo Sicco is a consultant for Pfizer and Aquis. Drs Desai, Nohria, Alhanshali, and Buontempo have no conflicts of interest to declare.
